# A Graphene-Based Flexible Pressure Sensor with Applications to Plantar Pressure Measurement and Gait Analysis

**DOI:** 10.3390/ma10091068

**Published:** 2017-09-11

**Authors:** Cunguang Lou, Shuo Wang, Tie Liang, Chenyao Pang, Lei Huang, Mingtao Run, Xiuling Liu

**Affiliations:** 1 Department of Biomedical Engineering, College of Electronic Information Engineering & Key Laboratory of Digital Medical Engineering of Hebei Province, Hebei University, Baoding 071002, China; loucunguang@163.com (C.L.); wangshuo5970@sina.com (S.W.); lanswer@163.com (T.L.); pancheyapcy@sina.com (C.P.); 2 Department of Molecular, Cell and Cancer Biology University of Massachusetts Medical School, Plantation Street, Worcester, MA 01605, USA; lionishystone@gmail.com; 3 College of Chemistry & Environment Science, Hebei University, Baoding 071002, China; lhbx@hbu.edu.cn

**Keywords:** graphene, flexible textile, pressure sensor, foot plantar, gait analysis

## Abstract

In the present study, we propose and develop a flexible pressure sensor based on the piezoresistive effect of multilayer graphene films on polyester textile. The pressure response results from the deformation of graphene conductive network structure and the changes in resistance. Here, we show that the graphene pressure sensor can achieve a sensitivity value of 0.012 kPa${}^{-1}$, the measurement range can be as high as 800 kPa, and the response time can reach to 50 ms. Subsequently, a stable in-shoe wireless plantar pressure measurement system is developed and dynamic pressure distribution is acquired in real-time. Overall, the graphene textile pressure sensor has the advantage of wide dynamic range, flexibility and comfort, which provides the high possibility for footwear evaluation, clinical gait analysis and pathological foot diagnosis.

## 1. Introduction

The foot is the terminal link of the kinematic chain in human locomotion and it experiences repetitive stresses from bearing the weight of the human body on a daily basis. Furthermore, the interactive forces are transferred between the human body and the ground during walking. The human foot experiences the pressure on the foot skin during daily activities, which means plantar pressure. Therefore, many physical parameters can be obtained from the distribution analysis of plantar pressure, and it has been employed in many areas, including footwear design, balance ability evaluation, rehabilitation training and gait analysis [1,2,3]. The evaluation of a diabetic subject’s foot is another important application of plantar pressure measurement, which may help to reduce the risk of ulcer recurrence in patients with diabetes [4]. The plantar pressure can be obtained by footprint method, force plate, pressure shoes and insoles [5,6]. Force plate is the most widely used instrument for plantar pressure measurement, but it is generally used in a static situation. Using the pressure insole can overcome the disadvantage of force plate, because the in-shoe systems can fit the foot and allow the measurement under various conditions without causing inconvenience and discomfort to the wearer.

Sensors with high sensitivity, reliability, flexibility and fast response, can be of great help for the popularization of health care devices and enhance the quality of life of users. To develop pressure shoes and insoles, many researchers are working on the innovation of pressure sensing elements to improve the wearing comfort [7,8]. Pressure-sensitive materials based on mechanism of capacitive sensing, piezoelectric sensing [9,10] and piezoresistive sensing [11,12] have been reported. The piezoresistive sensors have attracted much attention due to their feasible preparation and easy collection of signal. Currently, great progression has been made in the design of piezoresistive pressure sensors. For example, a pressure sensor based on flexible conductive sponges has been reported and applied in electronic skin [13], textile sensor with silver coated polyester yarns was developed for plantar pressure measurement [14], and graphene nanoplatelets and carbon nanotubes were screen printed on substrates to develop flexible resistive pressure sensor [15]. Polycrystalline silicon [16,17], micromachined ceramic and microstructured rubber dielectric layers [18,19] were also employed for generation of excellent sensors. Meanwhile, graphene is a highly promising material for development of piezoresistive pressure sensor because the changes of graphene in the resistance can be observed when loads applied [15,20]. Moreover, graphene has superior properties in biocompatibility, elasticity and stiffness [21,22,23,24], which makes it an excellent candidate material for flexible wearable sensors. Fu et al. fabricate the strain sensor devices using chemical vapor deposition (CVD)-grown graphene on transparent flexible polydimethylsiloxane (PDMS) substrate [25], while the fabrication process is rather complex and the graphene film is easy to fall off when contact with skin. Tian et al. developed flexible and ultra-sensitive resistive pressure sensor with a foam-like structure based on laser-scribed graphene (LSG) [26], while the dynamic response range (50 kPa) is not large enough for plantar pressure measurement.

In the current study, we generated a pressure sensor with graphene textile, which was synthesized by reduction of graphene oxide with the help of vacuum filtration. The pressure response resulted from changes in intrinsic resistance of the sensor, which were induced by deformation of graphene network in textile fibers. Subsequently, the developed graphene textile pressure sensor was embedded in an insole and a wireless plantar pressure measurement system was developed. Finally, the real-time plantar pressure distributions were obtained and used for quantitative gait analysis.

## 2. Experimental Section

### 2.1. Materials and Measurement System

The flexible pressure sensor was made by adhering conductive graphene films on the upper and bottom of textile fabric. Water-dispersed graphene oxide (GO) with a concentration of 4–6 mg/mLwas acquired commercially from XFNANO Materials Tech Co., Ltd (Nanjing, China). Dried white polyester fabric (200 D, 5 cm diameter) was obtained from cloth store. The diameter of single polyester fiber is 0.10 mm, the yarn linear density is about 110 per inch, and the thickness of the fabric is about 0.22 mm. Through dipping in GO solution and vacuum filtration, the polyester fabric was wrapped with GO, and then it was heated in an oven at 40–80 ${}^{{}^{\circ}}$C for 3–10 h. After heating, the three dimensional textile composite structure with GO was reduced by hydrazine hydrate (Alfa Aesar, Beijing, China) with a concentration of 0.15–0.20 mol/L [27,28]. Finally, the material was washed with water and dried at 40–80 ${}^{{}^{\circ}}$C for 3–10 h, then the three-dimensional multi-layer structure graphene textile was obtained.

**Figure 1:**
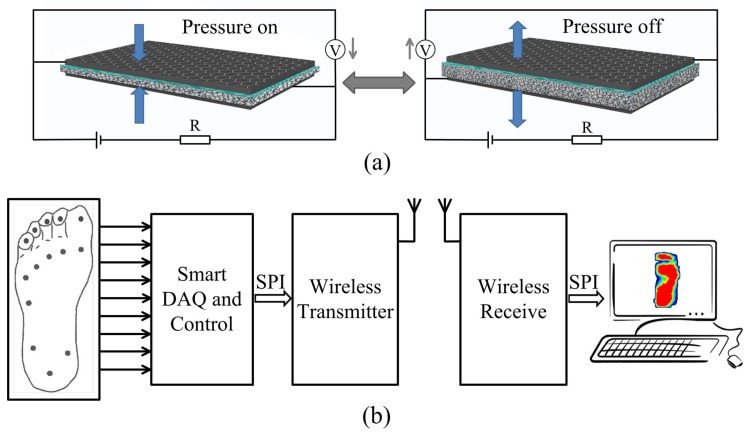
(**a**) The working principle of pressure-sensitive graphene textile sensor. The flexible textile acts as the support substrate and graphene layers wrapped around each polyester fiber. The thickness of textile decreases with the increasing applied pressure. (**b**) Block diagram of the proposed plantar pressure dynamic measurement system.

Figure 1a showed the working principle of pressure-sensitive sensor generated by one piece of graphene textile. The graphene are sticking on the surfaces of polyester fibers, and the structure deformation of flexible textile occurred when a force is applied, resulting in an increase of contact area in the polyester fibers, which would cause the sensor to experience a decrease in intrinsic resistance. When the pressure is released, the polyester fiber structure could recover their initial status. The sensitivity of deformation can be controlled by employing substrate material with different elastic modulus. A constant voltage of 3.3 V was applied to the series circuit of graphene textile and resistance R, and the resistance variation of graphene textile was converted to alternating voltage by the resistor voltage divider.

Figure 1b showed the block diagram of data acquisition system. We implemented pressure sensors array on an insole which was consist of 14 graphene sensors with 0.8 cm${}^{2}$ area for each one and conductive metal sheets were pasted on the upper and lower surface of graphene textile to make electrical contact with the wires. Firstly, the pressure related voltage signal was obtained and amplified with an inverting amplifier and sent to the data acquisition (DAQ) system for digitizing under control of the STM32 microprocessor (STMicroelectronics, Geneva, Switzerland). Next, the data was connected to radio frequency (RF) wireless transmitter/receiver module (NRF24L01+, Nordic Semiconductor, Trondheim, Norway) with serial peripheral interface (SPI). Finally, the data was collected and the pressure distribution image was obtained and displayed in PC. The data sampling rate is 30 Hz and the maximum transmission distance of wireless transmitter module reaches up to 150 m.

### 2.2. Characterization by SEM, Raman and XRD Spectroscopy

**Figure 2:**
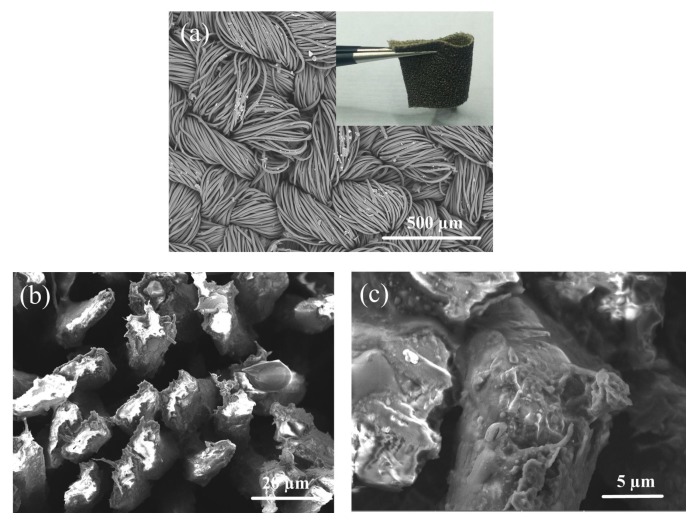
(**a**) The photograph of graphene textile sensor under low magnification. (**b**) The cross-sectional SEM image of graphene fibres at 3000× magnification. (**c**) at 10,000× magnification.

The morphological structure of graphene fibres was observed using TM3030 (Hitachi High-Tech, Tokyo, Japan) scanning electron microscope (SEM) and representative pictures are shown in Figure 2a–c. The photograph of graphene textile was shown as inset in Figure 2, which exhibited excellent flexibility. As shown in Figure 2a, polyester fibers were interlaced into a thicker line in a fixed way and some stack-like morphology can be seen. These small aggregates were formed because of the extremely high specific area of GO and the strong particle-matrix interactions. In contrast, a relatively homogeneous dispersion of the reduced GO was observed on the surface of polyester fibers thread in Figure 2c. Figure 2b shown typical cross sectional SEM images of graphene textile with a polyester fibres core of 5–16 μm diameter and a graphene sheath of about 1.5 μm thickness.

**Figure 3:**
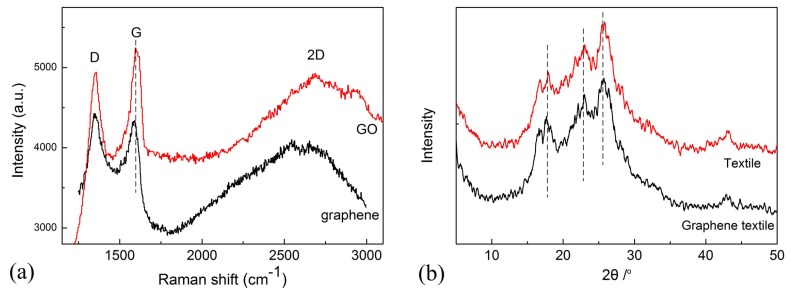
(**a**) Raman spectra of graphene textile before (GO) and after reduction (graphene). (**b**) X-ray diffraction (XRD) curves of polyester textile and graphene textile.

The graphene textile was also characterized by Raman spectroscopy using a Horiba Jobin Yvon confocal LabRAM HR800 spectrometer (Longjumeau, Paris, France). As shown in Figure 3a, both GO and reduced GO (graphene) revealed pronounced peaks at 1350 cm${}^{-1}$ and 1590 cm${}^{-1}$, which corresponds to the D-band and G-band, respectively. After reduction, the G band undergoes a small down-shift to that of graphite (1580 cm${}^{-1}$), suggesting a recovery of the honeycomb network of carbon atoms [11]. The prominent D peak with intensity comparable to the G peak along with their large band width is indicative of significant structural disorder in graphene textile. The D/G intensity ratio has been used as a measure of the average size of the sp${}^{2}$-domains [27]. As present in this figure, the D/G ratio in graphene (1.02) was found to be slight larger than GO (0.94), indicating a decrease in the average sizes of the sp${}^{2}$-domains, because the new sp${}^{2}$ domains in graphene are smaller in size compared to that in GO [11]. Weak and broad 2D peaks were another indication of disorder, in a single layered graphene, I${}_{2D}$ was greater than I${}_{G}$, whereas in a bilayer graphene, both were almost equal [29,30,31]. Figure 3a indicated that the 2D band of the graphene textile was centered around 2600–2700 cm${}^{-1}$, corresponding to the characteristics of multilayer graphene sheets.

X-ray diffraction (XRD) measurements were performed directly on the sample of polyester textile and graphene textile by an X-ray diffractometer (D8 ADVANCE, Bruker, Germany) using CuKα radiation (λ = 0.15418 nm) with 30 kV of voltage and 30 mA of current. The scanning rate was maintained at 0.1${}^{{}^{\circ}}$ s${}^{-1}$ over a 2θ range of 5–60${}^{{}^{\circ}}$ and the results were shown in Figure 3b. The diffraction curves of polyester textile and graphene textile are typical pattern of the polyester fabric [32], which exhibit 4 characteristic peaks at 2θ value of 17.9${}^{{}^{\circ}}$, 23.0${}^{{}^{\circ}}$, 25.8${}^{{}^{\circ}}$ and 43.1${}^{{}^{\circ}}$, respectively. Here it is hard to find the characteristic peak of graphene in the patterns of modified polyester textile due to the low amount of graphene loaded. The characteristic peaks of GO at 2θ value of 11.72${}^{{}^{\circ}}$ can not be obtained, indicating the successful reduction of oxygen functional groups.

## 3. Results and Discussion

**Figure 4:**
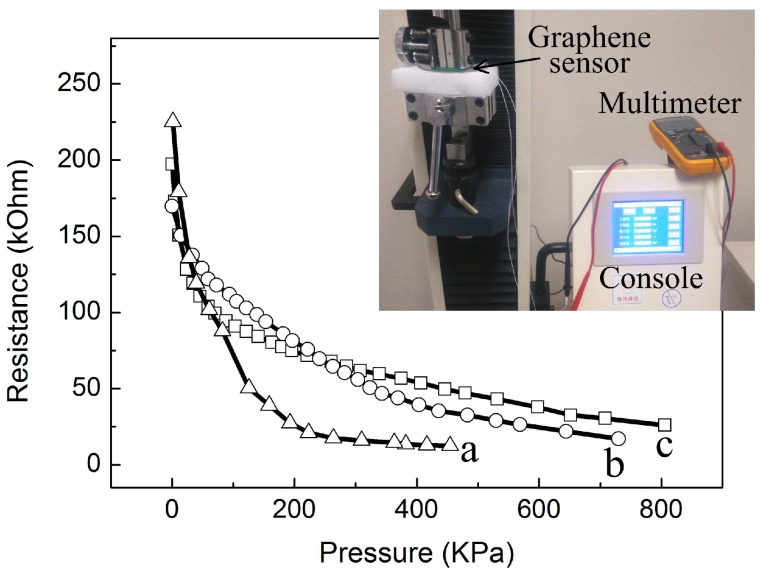
The resistance curves of the graphene textile pressure sensors vs. applied loading. a: 2-layers textile sensor; b: 3-layers textile sensor; c: 4-layers textile sensor.

In the test procedure, a digital push gauge (QX-W600, Shanghai Qixiang Industrial Co. Ltd., Shanghai, China) was used to provide the required pressure. Conductive copper tapes were pasted on the upper and lower surface of graphene textile and a digital multimeter (Fluke, Everett, Washington, USA) was used to monitor the change in resistance of graphene textile between two copper tapes. To illustrate the influence of textile layers on the sensitivity, different pieces of graphene textile were employed to construct pressure sensors. The 2, 3 and 4-layers textile sensor were assembled by stacking 2, 3 and 4 pieces of graphene textile, respectively. Measurement was performed on the 3 sensors in sequence and the pressure responses of them were present in Figure 4. As shown in the figure, when the applied load was increased, the resistance of those sensors was significantly reduced, changing from about 200 kΩ to thousands of ohm. Furthermore, a negative exponential decay function can be obtained and the nonlinear response can turn to linear response by using a logarithmic transformation circuit. While, approximate linear response with different sensitivity in 3 separate measurement intervals can also be obtained. For the 2-layers textile sensor, the high-sensitivity response range was from 0 to 50 KPa, and it tended to become saturated when the pressure reached to 230 KPa. Therefore, the cut-off point of those 3 segments was 50 and 230 kPa, respectively. Similar response curves can also be obtained for 3 and 4-layers textile sensors, and the cut-off point was 60 and 340 KPa, 35 KPa and 180 KPa, respectively. The different sensitivity of those 3 sensors may result from different amount of graphene films attached on the textile.

The sensitivity S can be defined as S = δ(ΔR/R${}_{0}$)/δp, where δ is the differential operator, ΔR is the resistance change upon applied pressure of P, and R${}_{0}$ is the initial resistance without applied pressure [20,22]. In the high-sensitive response interval, the sensitivity value of the 2-layers textile sensor reached 0.012 kPa${}^{-1}$, and the sensitivity value of 3 and 4-layers textile sensors were 0.0017 kPa${}^{-1}$ and 0.0048 kPa${}^{-1}$, respectively. The shape variability of supporting materials directly affected the three-dimensional structure of graphene sensor, suggesting that employing elastic sponge as the supporting materials will greatly improve sensitivity [12]. As shown in the figure, more textile layers resulted in larger dynamic response range. The measurement range of 2-layers textile sensor reached 450 kPa while it reached 800 kPa when employing the 4-layers textile, this observation was closely related to the compressibility of textile substrate. The sensor was dramatically deformed when pressure was loaded, leading to a rapid increase in contact area of polyester fibers with conductive graphene. This caused the resistance to sharply drop, whereas the textile deformation tended to saturation due to its relatively high compactness. Employing more pieces of textile led to an increase in increased compression of flexible substrate, and the measurement range will be dramatically enhanced.

**Figure 5:**
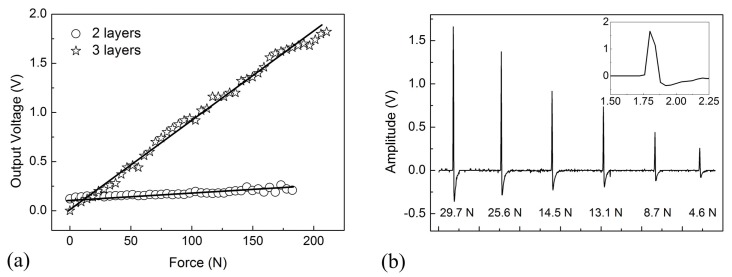
(**a**) Changes of output voltage as a function of stepped load for the designed graphene textile pressure sensor. (**b**) The output changes of the sensor at different loading values. The insert showed the waveform at a load of 29.7 N.

An effective pressure sensor must possess not only high linearity and sensitivity, but also rapid response and high reliability. To investigate the linearity of the graphene textile sensor, a series of loads were applied on the sensor by adjusting the free-fall height of a 520 g steel ball. The process was repeated five times and the average value was obtained. Moreover, the influence of textile layer number on the sensitivity was also studied. As shown in Figure 5a, the output voltage displayed an obvious increase as external pressure increases, and the relative linearly pressure response was obtained. Meanwhile, the 3-layers textile sensor exhibit higher sensitivity due to the more severely deform and greater change in contact area. A series of pressures ranging from 4.6 to 29.7 N were applied and released on the 2-layers textile pressure sensor, and the regular bipolar waveforms output was illustrated in Figure 5b. It can be noted that the output amplitude increased along with the rise of loading force. From the inset of Figure 5b, an instantaneous voltage increase with a response time of only 50 ms was observed, serving as evidence for the fast response of the sensor.

**Figure 6:**
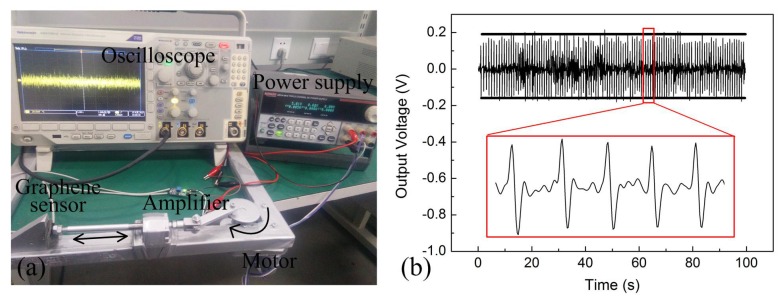
(**a**) Laboratory set-up for the study of the reliability of graphene sensor. (**b**) Repeated measurement of the sensor to evaluate its sensing stability: 120 cycles of 50 kPa applied at 1-s intervals. The insert showed detail of 5 cycles.

To further illustrate the reliability of the sensor, a pressure of 50 kPa was alternately loaded and unloaded onto the sensor for 120 cycles by a reciprocating DC motor (Mabuchi, Japan), and the results were displayed in Figure 6. Fairly constant output voltage throughout testing was obtained with the application of pressure loads, indicating a good stability and repeatability of the graphene textile sensors. The details of 5 cycles were also given, the rising edge and falling edge of captured signals corresponded to the application and removal process of pressure load, respectively. As can be seen in the detail figure, the magnitude of output voltage fluctuation was less than 10% and the slight fluctuations may resulted from the instability of sample platform.

**Figure 7:**
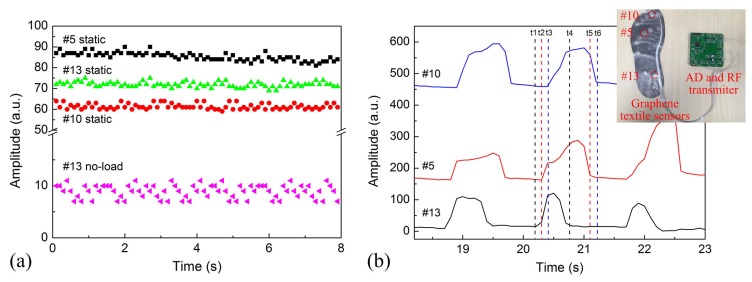
(**a**) The pressure amplitude of 3 sensors in the static standing state. (**b**) A stride of raw signals measured from right foot of an able-bodied subject.

The pressure insole with 14 graphene textile sensors was designed and the distribution of these 14 sensing elements supported most of the body weight and adjusted the body balance. In the experiment, the subject was 24 years old, 70 kg in weight and 1.72 m in height. To elucidate the stability of the pressure measurement system using graphene textile, the dynamic pressure came from 3 sensors were collected respectively in the static standing state. Figure 7a showed the varieties of the pressure amplitude of the sensor #5, #10 and #13 in the static standing state, and signals in the no-load state were also given for comparison. The standard deviation reflected the degree of discretization of a data set and the degree of deviation from the mean. In the no-load state, the mean values and standard deviations of the pressure amplitude of sensor #13 was 8.91 ± 1.28. In the static standing state, the mean amplitudes and standard deviations of the signals measured from sensor #5, #10 and #13 were 85.3 ± 1.95, 61.4 ± 1.36, and 71.9 ± 1.28 and average signal variance ratio (the ratio of standard deviation to mean value) was 2.29%, 2.21%, and 1.78%, respectively. No obvious signal drift over time was observed, indicating the relative stability of the proposed insole plantar pressure measurement system. The difference in their amplitude resulted from different plantar pressures of fore-foot and rear-foot regions.

The pressure amplitude and raw signals from 3 sensors were obtained during normal walking trial and were shown in Figure 7b. In addition, we noticed that the signals measured by the 3 sensors during walking had different amplitudes and exhibited a periodicity. Different pressure not only effectively reflected pressure values in the pressure insole measured at different times, but also provided the information for describing gait characteristics and performing gait analysis. The pressure amplitudes of the 3 sensors at different time points correspond to the foot fall on and off the ground during walking. As can be extracted from the time series data, the rear-foot landing on ground firstly, then the mid-foot and rear-foot landing with a 104.8 ms and 215.5 ms time delay, respectively. The landing time duration of fore-foot, mid-foot, and rear-foot in a gait cycle was 765 ms, 992.2 ms and 1004 ms, respectively. The stance phase accounted for 72.2% of the total gait cycle, and the swing phase comprised the remaining 27.8% of the gait cycle. In addition, the maximum mean peak pressure of the fore-foot (MP-F), mid-foot (MP-M) and rear-foot (MP-R) was around 150, 133 and 145 (a.u.) respectively. The shift speed of the center of pressure (COP) was an important parameter to assess static and dynamic balance abilities of an individual. When the shift speed of COP exceeded a certain threshold, meaning that the person was not walking in a stable and balanced way [12]. For the volunteer, the shift speed of COP was calculated to be 267.7 mm/s. Detailed description of the participant characteristics is shown in Table 1.

**Figure 8:**
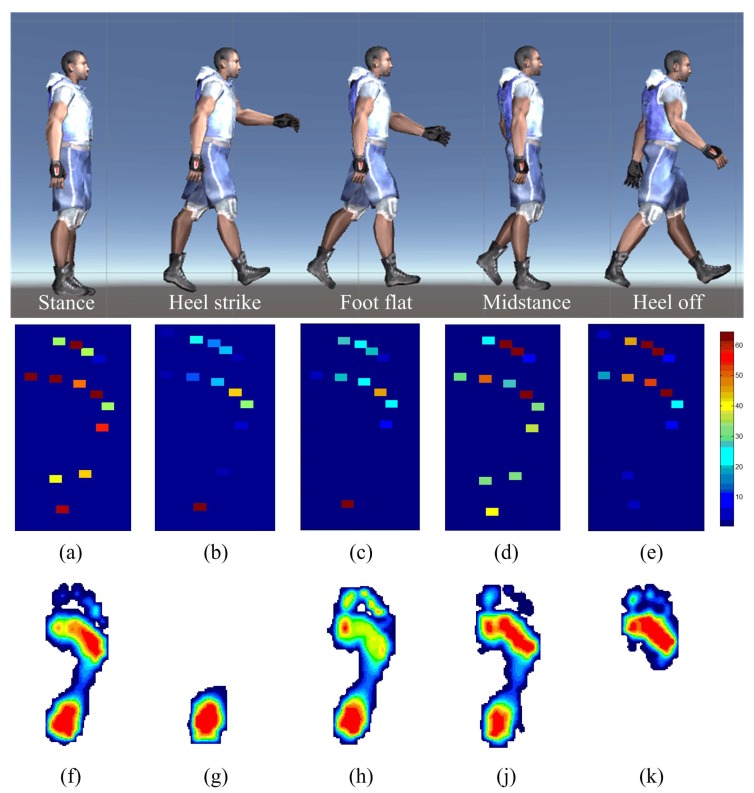
Plantar pressure distribution of right foot in the major phase of one full gait cycle. (**a**–**e**) images measured by our developed system. (**f**–**k**) images obtained by commercial pressure measurement device.

Gait is one of the most frequently used forms of human movement during daily activity, because it directly affects balance and stabilization of the body during movement. The gait cycle is fundamentally divided into stance and dynamic swing phases. Plantar pressure during the major phases and events of a full gait cycle was measured by the developed portable system and the distribution maps were displayed by a color legend ranging from dark blue (low pressure) to red (high pressure) in Figure 8a–e. The reliability of the system was assessed by a commercial Matscan system (Tekscan, Boston, MA, USA). As present in the figure, a gait cycle typically consists of stance, heel strike, foot flat, midstance and heel off. In the static standing state, the weight of the body was evenly distributed over the whole foot, which was shown in Figure 8a,f. During the dynamic swing phases, from rear-foot landing on the ground to forefoot landing off the ground, the body weight was transferred correspondingly. The mean pressure value on fore-foot region obtained from Figure 8b–e was 15.2, 13.4, 61.7 and 69.1, respectively, and the mean pressure value on rear-foot region was 45.6, 37.9, 76.0 and 29.3, respectively. Correspondingly, the forefoot-to-rearfoot plantar pressure ratio was increased from 0.3 to 2.35 and an overall good agreement with those obtained from Matscan system (Figure 8g–k) was observed. It can be noted that, the plantar pressure in Figure 8d and fore-foot region in Figure 8e were much larger than that in other figures. The variation was due to the right foot, which came into stance phases and suffered from the majority of the body weight.

## 4. Conclusions

In summary, we have demonstrated a pressure sensor based on a multi-layer graphene taking textile fabric as the support body. The use of soft textile substrate allows applied pressure to be converted into structural deformation that is then delivered to the graphene layer, generates resistance changes and is converted into electrical signal output. In our study, the sensitivity value is up to 0.012 kPa${}^{-1}$, the signal has good reproducibility in response to different pressures, and the sensitivity of the sensor can further increase by the amount of control of the graphene films. The measurement range can be as high as 800 kPa, corresponding to the range of human pressure perception for the recognition of body signals. Compared with most of the reported highly sensitive pressure sensors, whose measurement range was lower than 100 kPa, our pressure sensor gets good results in a wide measurement range with an acceptable sensitivity, while it is sufficient for the application of plantar pressure measurement. The characteristics of high-sensitivity and dynamic range, good repeatability, flexibility, and comfort provides the high possibility to fit on various wearable applications, such as monitoring daily activity and exercise with motion, assessing the balance and gait disorders in older adults and outpatients, and monitoring the pulse and respiratory waves.

## Figures and Tables

**Table 1:** The participant characteristics were obtained during a walking trial.

Characteristics	Value	Characteristics	Value
Gait cycle	1.51 s	MP-F	150
Stance phase	72.2 %	MP-M	133
Swing phase	27.8%	MP-R	145
Shift speed of COP	267.7 mm/s		
